# Contributors to Delayed Prenatal Care among Rural and Underserved Populations: A Qualitative Study

**DOI:** 10.21203/rs.3.rs-7456155/v1

**Published:** 2025-09-30

**Authors:** Andrea Lara Research, Oluwatobi E. Oladeji, Julie A. St. John, Jennifer Schiele, Casie Stoughton, Stephanie Stroever, Teresa Baker, Christine D. Garner

**Affiliations:** Texas Tech University Health Sciences Center School of Medicine, Amarillo, Texas, United States; Texas Tech University Health Sciences Center School of Medicine, Amarillo, Texas, United States; School of Health Professions Texas Tech University Health Sciences Center, Lubbock, Texas, United States; Texas Tech University Health Sciences Center School of Medicine, Amarillo, Texas, United States; Amarillo Public Health District, Amarillo, Texas, United States; Texas Tech University Health Sciences Center School of Medicine, Amarillo, Texas, United States; Texas Tech University Health Sciences Center School of Medicine, Amarillo, Texas, United States; Texas Tech University Health Sciences Center School of Medicine, Amarillo, Texas, United States

**Keywords:** pregnancy, prenatal care, delayed care, access to care, rural health, insurance, transportation, health literacy, qualitative methods

## Abstract

**Introduction:**

Timely and adequate prenatal care is critical for improving pregnancy outcomes. Rural and underserved women in the U.S. have a higher burden of chronic disease, higher risk of adverse pregnancy outcomes, and lower access to maternity care services. This study explored perceived barriers and challenges to prenatal care in rural and underserved counties in the Texas Panhandle.

**Methods:**

Semi-structured qualitative interviews were conducted with 34 key informants (maternal health partners, healthcare professionals, and organizational leaders) in six Texas Panhandle counties. Recruitment included purposive and chain-referral sampling for maximum variation. Thematic analysis was conducted using ATLAS.ti (Version 25.0.1); coding was emergent and iterative. Findings were structured around major themes identified within the Social Ecological Model.

**Results:**

Delayed entry into prenatal care emerged as a significant overarching problem driven by four main barriers: 1) lack of insurance at the start of pregnancy resulted in delays in coverage and thus limited early access to care; 2) healthcare setting barriers included policies and practices that delayed care for uninsured women and too few providers for rural and low-income women; 3) long distances and limited transportation options, whether in rural or underserved urban areas, impacted the ability to attend prenatal care visits; 4) gaps in knowledge about insurance, appropriate timing of prenatal care, and available community resources also delayed healthcare engagement. These barriers were exacerbated by community-based beliefs and challenges related to low-income, resulting in transportation difficulties, constraints in accessing online insurance applications, and the inability to afford out-of-pocket healthcare.

**Conclusion:**

Community-driven strategies to improve early entry into prenatal care in these rural and semi-urban areas include addressing insurance enrollment, education, healthcare setting barriers, and distance to resources and care. Understanding and addressing these barriers among rural and underserved communities may improve the timeliness and adequacy of prenatal care which is crucial for identifying and managing maternal health conditions and preventing complications.

## Introduction

Prenatal care is a preventative service that plays a critical role in the well-being of pregnant individuals and their children. The American College of Obstetricians and Gynecologists (ACOG) recommends starting prenatal care in the first trimester, preferably 8 to 10 weeks of gestation, followed by 12 to 14 visits during a full-term pregnancy^(1)^. It is well-established that prenatal care education has been associated with increased breastfeeding rates, improved newborn care knowledge, enhanced maternal self-efficacy and reduced fear and anxiety related to childbirth^(2)^. Unfortunately, national data for the United States (U.S.) show that nearly a quarter of women began care later than recommended, with 17% initiating care in the second trimester, 4.7% in the third trimester and 2.3% receiving no care at all^(3)^. The timing and adequacy of prenatal care are widely recognized as important factors in maternal health outcomes.

Disparities in maternal morbidity and mortality are evident in both rural and urban areas across the U.S. Notably, the U.S. continues to report higher rates of maternal morbidity and mortality than any other high-income country^(4)^. Data from a study using the Pregnancy Mortality Surveillance System (2011–2016) showed that pregnancy-related mortality ratios were 1.5 to 3 times higher in rural compared to urban areas^(5)^. Furthermore, between 2016 and 2019, maternal mortality in rural areas increased from 74.6 to 81.7 deaths per 100,000 live births, while urban areas also saw a rise, albeit a smaller one, from 40.2 to 42.3 per 100,000^(6)^. While rural communities continue to face disproportionately high rates, the upward trend in urban areas signals a broader national concern that extends beyond geographic boundaries.

Access to prenatal care is complicated for rural populations who face unique challenges that contribute to differences in maternal health outcomes. Maternity care deserts are defined as counties where there is no access to birthing hospitals, birth centers offering obstetric care, or obstetric providers^(7)^. Between 2006 to 2020, more than 400 maternity care services closed across the United States. A recent cross-sectional study found that maternal mortality rates in maternity care desert counties were significantly higher, 32.25 per 100,000 live births, compared to 23.62 in counties with full access to care^(8)^. Up to 40% of U.S. counties have been designated as maternity care deserts. In 2022, 2.2 million women of reproductive age lived in counties that lacked access to maternal care^(8)^. These women tended to have poorer preconception health, were less likely to receive timely prenatal care, and experienced higher rates of maternal mortality and pregnancy-related mortality^(8)^.

In addition to geographic barriers, social determinants–economic stability, education, healthcare access, transportation, environmental conditions, and social and community context–influence health and the ability to seek and access care. These system-level barriers are deeply ingrained in the social conditions in which people live, work, and interact, compounding the effects of geographic isolation on maternal health^(9)^. Health insurance status is one such barrier. In the U.S., an average of 9.7% of women aged 18–64 are uninsured. Rates vary widely by state, from 2.6% in Massachusetts to 20.7% in Texas^(10)^. Understanding whether, how, and among whom system-level barriers are experienced may help identify contributors to differences in prenatal care and opportunities for improving access.

Access to prenatal care is paramount in identifying and managing problems during pregnancy, monitoring the growth of the fetus, minimizing risks of preterm birth or low birth weight and its complication, and maternal mortality. A maternal health coalition in a largely rural area in Texas sought to improve maternal health outcomes by expanding access to care and equipping women with essential knowledge and resources. The coalition conducted a needs assessment to identify perceived barriers and challenges contributing to maternal health challenges. This study aimed to identify and understand perceived barriers to prenatal care among community and healthcare leaders with the goal of creating sustainable, community-driven solutions to inform future implementation of an evidence-based maternal health intervention.

## Methods

### Study setting

The study team conducted this research across six counties (two urban and four rural) in the Texas Panhandle. Population-weighted racial/ethnic composition for the people across these counties are White (86%), Hispanic (42%), Black (6%) and other races (6%) (U.S. Census Bureau, 2024). The primary languages spoken are English and Spanish; however, more than 30 languages are spoken in these counties due to the large groups of individuals from Central and South America, the Caribbean, Africa, and Asia. Numerous residents speak Spanish only or other dialects such as Quiché/K’iche’. The six counties selected for this study have known disparities in maternal health and limited or nonexistent access to maternity care. This study was part of a community needs and resource assessment to understand maternal health needs in this region. A community-engaged research Coalition of 30 local organizations guided this project, including the development of questions to be asked in interviews and identification of people and organizations to be included. Key informants for the study included maternal health stakeholders and opinion leaders embedded within these communities.

### Recruitment and enrollment

Participants were recruited using purposive and chain-referral sampling methods, focusing on members of a maternal health coalition and individuals identified by coalition members. Recruitment materials clearly outlined the study’s aim of understanding maternal health challenges to inform strategies for improving care. Potential participants were thoroughly informed about the study’s purpose and the researchers’ roles, ensuring transparency throughout the recruitment process. This study was approved by the Texas Tech University Health Sciences Center Institutional Review Board, protocol # IRB-FY2024–107.

Recruitment strategies included face-to-face interactions, chain referrals, and outreach by coalition members including through a regional listserv. Interested individuals completed an electronic screening survey, which also collected basic demographic information. Eligible participants were contacted by research staff to confirm eligibility, answer any questions, offer an incentive choice and schedule participation in a key informant interview.

### Informed consent

was obtained electronically using Adobe Sign or using paper forms. Participants received the consent form and a video-recorded explanation via email, or the form was reviewed with them in-person. All consent documentation was securely stored.

Inclusion criteria required participants to be 15 years of age or older, speak English or Spanish, and either reside or work in one of the six Texas Panhandle counties–Deaf Smith, Gray, Parmer, Potter, Randall, or Swisher–or be affiliated with the coalition. Eligible participants included healthcare providers, social service providers, and leaders of organizations whose activities reached pregnant and postpartum women, and informal community leaders. Individuals were excluded if they were under 15 years of age, not residents of the specified counties, unaffiliated with the coalition, or unable to communicate verbally or in writing.

### Data collection/interview procedures

A semi-structured key informant interview guide was developed using the Consolidated Framework for Implementation Research (CFIR)^(11)^ and the Social Ecological Model^(12)^ Throughout the tool development process interview guides were sent out to coalition members for input and further refinement. The interview guide was tested and modified. (Table 1).

Interviews were conducted by research team members trained in qualitative interviewing techniques. To ensure convenience and accessibility, interviews were scheduled in private locations selected by participants, including their workplaces, community centers, schools, churches, local health department sites, and other public facilities. To accommodate those unable to attend in person, virtual sessions were also conducted via Zoom. Interviews were audio-recorded for in-person sessions, and both audio- and video-recorded for virtual interviews conducted via Zoom.

Data were collected from March 2024 to March 2025. Seventeen interviews were virtual, and seventeen were conducted in-person. Minimal interruptions were experienced. One interview was conducted with a director who had an employee present at the preference of the participant, and one interview was conducted with 2 key informants simultaneously, also at the preference of the participants. There were 34 interviews conducted in total with at least four interviews in each county. Field notes documenting contextual details and key observations were completed immediately after each session. Interviews lasted an average of 51 minutes (range: 13–102 minutes). After 24 interviews, data saturation, defined as the point at which no new information emerged, was reached.

### Data analysis

Qualitative analytic methods were employed in this study, with data collection and analysis occurring concurrently to allow for the exploration of emerging concepts throughout the study. Thematic analysis was carried out to identify concepts that arose from the data^(13)^. Transcripts were read alongside field notes to understand context, and a preliminary set of codes was developed based on the first 8 transcripts. Codes emerged from the data, and were added and iteratively refined as data collection progressed. Two coders used ATLAS.ti (Version 25.0.1) to support the coding process, with themes derived inductively. Independent coding was conducted by multiple team members, and team members met regularly to discuss new concepts that arose in the data, resolve discrepancies, and reach consensus. Over time, key themes and subthemes were identified by grouping concepts and codes that emerged.

Peer debriefing took place throughout the analysis, and findings were shared with coalition members to provide feedback and validate results–a process known as member checking^(14)^. Qualitative analysis was led by team members with prior experience in these methods, with support from additional trained research personnel.

The Social Ecological Model (SEM), was used to examine how multilevel barriers contribute to maternal health challenges^(12)^. This model considers the interplay between individual, interpersonal, institutional, community, and policy-level factors, offering a holistic perspective on how systemic, socioeconomic, geographic, and cultural barriers delay or prevent timely access to prenatal care. This model guided the identification of barriers as they emerged from the data.

## Results

### Participants

Key informants (n = 34) participated in the study, including 29 women and 5 men (Table 2). Participants ranged in age from 32 to 70 years; most identified as White (n = 22), 8 as Hispanic, 3 as Black and 1 as Asian. Participants represented a range of professional settings across the six counties, including administrators, social service providers, community leaders, and healthcare professionals such as medical doctors, registered nurses, nurse practitioners, and a pharmacist. Between 4 and 10 participants were included from each of the 6 counties.

### Themes

**Figure 1:**
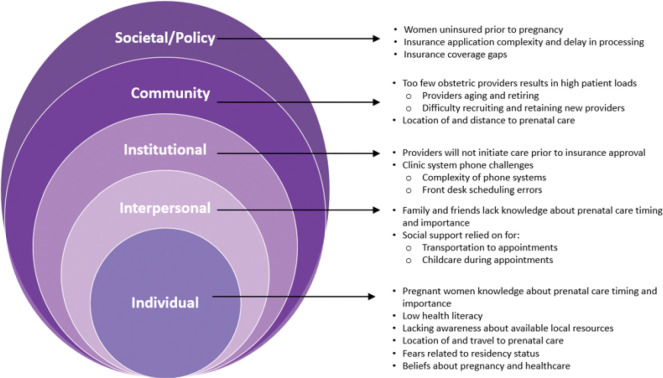
Perceived barriers to timely prenatal care across levels of the Social Ecological Model

The overarching theme that arose was delayed entry into prenatal care. Key informants described four main interconnected barriers that delayed or prevented timely access to prenatal care: (1) lack of insurance, (2) healthcare setting policies and practices, (3) long distances and limited transportation to providers, and (4) gaps in knowledge of prenatal care and community resources. These barriers were intensified by community-based beliefs, challenges, and income barriers such as transportation, difficulty accessing online insurance, and unaffordable out-of-pocket costs. Components of these themes spanned the Social Ecological Model (Fig. 1), and example quotations for each theme are provided in Table 3.

### Lack of insurance

Key informants consistently identified lack of health insurance as a major barrier to timely and adequate prenatal care. Many women in this region lacked health insurance prior to pregnancy, thus, applying for insurance upon discovering their pregnancy was necessary. Without insurance, self-pay for prenatal care visits was cost prohibitive for most women. The majority of the regional population was described as having low income; thus, public payer insurance (Medicaid) was the primary form of coverage, but applying for Medicaid was perceived to be a lengthy and difficult process. The application process was described as complex, slow, and poorly suited to applicants with limited digital literacy, internet access, or language proficiency. Because of this, assistance with Medicaid applications was needed:

“We don’t have enough Medicaid navigators to help people get on Medicaid when they need it.” KI103

Internet access was an additional hinderance to insurance applications. Unreliable broadband in rural and low-income areas and an inability to afford internet at home exacerbated these challenges. One key informant explained, “Probably economics. So that’s part of it and being able to afford [internet]” (KI116). These challenges contributed to incomplete or delayed applications and prolonged wait times for approval. Some participants highlighted that those with moderate incomes fell into insurance coverage gaps–earning too much to qualify for Medicaid but not enough to afford private insurance. As one informant noted:

“It’s getting to the point where it’s kind of like middle class [do not have insurance], because it’s too much [cost] to have insurance, but then it’s you don’t qualify for Medicaid… we’re seeing that trend of clients getting neglected because they don’t qualify.” (KI131)

Fear related to residency status was a recurring concern among participants when discussing insurance access. Specifically, it was described that some community members hesitate to apply for health insurance due to concerns about how their personal information might be used or shared with authorities. One key informant noted that many individuals:

“Do not feel like they will qualify… they’re afraid to apply because of their [residency status] or… they feel like they will be in trouble if they apply…” (KI108)

Altogether, not having insurance prior to pregnancy, barriers to applying for insurance, delays in processing of applications, and inability to pay for care in the absence of insurance resulted in delays in or absence of prenatal care.

### Healthcare setting challenges

Participants described policies that prevented prenatal care providers in the region from scheduling initial prenatal appointments prior to approval and proof of insurance. Thus, the insurance application and approval became critical steps that resulted in delayed entry into prenatal care.

“They’ve applied for Medicaid, but three months later, they still don’t have an answer… they haven’t had any prenatal care because no one’s gonna see them without insurance.” (KI103)

Furthermore, participants noted that most healthcare providers did not accept uninsured patients, and some accepted only privately insured patients, and not Medicaid. Thus, pregnant women with low income had few options for care providers.

“Lack of available doctors to be able to see them for the insurance that they do have. So if they have Medicaid, there’s a long wait.” (KI102)

A shortage of obstetric providers in rural and low-resource areas was a major barrier to accessing prenatal services. Practicing obstetricians were described as having high patient volumes and limited availability in scheduling; thus, resulting in delays in initiating care. Participants emphasized that obstetrics and prenatal care services have been steadily disappearing from these communities due to a dwindling provider workforce. Cited factors contributing to this included aging providers’ retirements, relocation for better pay, and ongoing challenges in recruiting and retaining obstetricians in rural areas. As one key informant explained,

“[Our rural] county does have maternal health [care]…very limited because a lot of the providers were older and a lot of them were leaving the practice… [another rural] county has not had maternal health for about 20 years now. Their last provider retired and they chose not to go that route.” (KI109)

Communication systems and channels were described as additional barriers within prenatal care clinics. Phone systems and front desk coordination were identified as barriers, including long hold times for patients calling to schedule appointments, and complicated navigations menus over the phone: “Sometimes I’ve called the front desk and I’m like… how many more numbers do I need to press… I think that’s a huge barrier for patients to get in” (KI123).

Poor communication channels between clinics and community organizations were also described as barriers to timely access to critical services for high-risk patients.

“There’s a big delay… if there was someone we could call and say I have… a six weeks, type one diabetic on insulin… She’s trying to keep her sugars under control, but she’s probably going to need her insulin adjusted and we can’t do that. So, can we get her in quicker?…That would be amazing to be able to do.” (KI110)

This administrative lag created a vulnerable window in which expectant mothers were without access to essential care, particularly in the first trimester, when early screening and interventions are most beneficial. Inefficiencies in communication and scheduling disrupted the timely delivery of care and posed additional barriers among patients in need of immediate attention.

### Distance and transportation to prenatal care

Women in both rural and low-income neighborhoods experienced challenges due to lack of proximity or long distances to care. Most of the rural counties had no prenatal care available, thus, participants described that women in these counties had substantial travel time, “more than two hours each way,” to attend prenatal care appointments. The travel time was described as burdensome:

“Going to [a larger city] is actually an all-day thing… you’ve just spent six hours going to one appointment and that takes your day.” (KI109)

Within cities, differences existed between neighborhoods, with clinics and hospitals frequently concentrated in or near areas with higher income or more resources. These challenges were exacerbated by inadequate transportation options. Pregnant women without a personal vehicle, relied on public or community transportation, which were scarce in rural areas. One participant expressed, “They’ve made Medicaid transportation so difficult to get. I have people start [an application] and they don’t finish because you have to have so many documents, so many they give up.” (KI111)

Long travel distances to care were exacerbated by rising fuel prices, placing additional financial strain on families already experiencing economic hardship. Participants described that some women coordinated rides with family or friends or delayed care altogether because of inadequate access to transportation to attend prenatal care visits.

“These families don’t always have the money… to pay for gas. They may not have a vehicle… they may have to rely on others to take them.” (KI104)

Lastly, other logistical challenges to accessing prenatal care included rigid job schedules, limited paid leave, and hesitancy to request time off for appointments. These challenges contributed to missed appointments or delayed care:

“I know that… one, she works out at [a factory] and they’re very restrictive on giving their employees like time off and things like that for appointments.” (KI107)

### Gaps in knowledge and understanding about prenatal care

Participants described community gaps in knowledge about appropriate timing of prenatal care and processes for obtaining health insurance. Participants believed that many pregnant women did not know when or why to seek prenatal services, especially during the critical first trimester. One key informant emphasized the need for early education.

“In certain populations, just even knowing that [prenatal care] is necessary… [we should] educate on the importance of prenatal care as early as possible.” (KI116)

At community and organizational levels, participants pointed to a lack of community-wide knowledge and pre-pregnancy education as a major barrier to maternal health. Participants underscored the need for education about available services, emphasizing that without awareness, individuals are unlikely to seek care as early as they should. As one participant explained:

“I don’t think that there is really anything available in terms of educating the woman prior to pregnancy about the importance of early prenatal care. I don’t think we have that resource.” (KI130).

These gaps in knowledge highlight a broader need for community-based health education efforts that empower individuals and families with the necessary information to access timely and appropriate care.

Health literacy levels vary among pregnant women, as one participant described:

“They are struggling with low education level itself leads to not really understanding health or what a doctor tells you, so poor health literacy.” (KI109)

It is important that health education is tailored to all levels of health literacy, and adequate time is taken to ensure understanding.

Participants particularly emphasized the importance of providing education for teens and pregnant adolescents. One key informant reflected on the loss of a once-effective program:

“In 2015…[our school district] withdrew their course called teen parenting in which any student that was pregnant was dumped into that class… I taught it at [high school] and so, I was the teen parenting teacher and so I would have nurses come in and physicians come in and Medicaid people… all the different representatives come in to speak to my girls. [Our school district] no longer offers that.” (KI105)

Community-based beliefs were also described as affecting how and when some groups engaged with healthcare, often shaping health behaviors and decisions about when or whether to seek care. One participant highlighted how community norms influenced prenatal care-seeking behavior: “As it relates to when patients start care… I have a [group within our community] who almost reliably and instinctively wait until their third trimester to establish care. I have never… had a patient [from this group] start care early” (KI130). This delay in care may reflect deeply rooted community-based practices and beliefs about pregnancy or barriers in navigating the healthcare system.

## Discussion

Lack of pre-conception health insurance was a major barrier to timely and adequate prenatal care in our study. This barrier precluded any preconception healthcare and resulted in delays in obtaining insurance and delays in initiating prenatal care. Data across our study counties show high proportions of uninsured women (ages 18–64) ranging between 14.5% and 30.6%^(15)^. Out-of-pocket costs for prenatal care exceeded what was financially feasible; thus, insurance was typically needed to attend a prenatal care visit. Women without insurance typically lack it either because they were unaware of their eligibility for public payer insurance (Medicaid), they were ineligible for Medicaid or CHIP prior to pregnancy, or because of the high cost of private insurance. Previous research confirms that lack of insurance is a leading determinant of delayed prenatal care. Difficulties with insurance coverage, including being uninsured and transitioning between coverage from the preconception to postpartum period, are associated with women being less likely to receive early prenatal care^(16)^.

Key informants described that most pregnant women across the region relied on public payer insurance (Medicaid) as their primary form of coverage. Applying for Medicaid was perceived to be a complicated process. Our findings are supported by previous research that showed navigating state-specific Medicaid policies were challenging and often followed by delays in enrollment approval^(17)^.

Through the Affordable Care Act, passed in 2010, states were given the opportunity to expand Medicaid eligibility to individuals with slightly higher incomes^(18)^. Texas is one of 10 states that has not expanded Medicaid; thus, it was not a surprise that a coverage gap was identified by key informants. The Medicaid gap is defined as people without private insurance who do not meet Medicaid requirements, which is 138% of the Federal Poverty Level or below. Lukens and Harker^(19)^ reported that approximately 726,000 adults in Texas fall in the Medicaid coverage gap. Research shows that in states where Medicaid was expanded there were improvements in perinatal care coverage and utilization, particularly in the first trimester^(20)^.

Health insurance status also influenced the timing of prenatal care due to policies and practices at prenatal care clinics. Study participants stated that clinics may hesitate to schedule visits until proof of coverage is received, even when applications are pending. Other studies identified that prenatal care providers have required insurance to schedule an appointment and/or limited the number of Medicaid-insured patients they accepted^(17)^. Retroactive coverage up to 90 days prior to active Medicaid enrollment is available in most states^(21)^, including Texas; however, this was not being utilized. Identifying and addressing such local healthcare organizations’ policies may be an important opportunity for improving entry to prenatal care.

Challenges with the process of enrolling in insurance were frequently described. Thus, strategies to assist pregnant women with enrollment are needed. Community health workers may be well-positioned to assist in insurance enrollment as outreach agents. Community health workers often share lived experiences and norms with their communities, they build trust, coordinate care, serve as effective health educators, and bridge healthcare settings with communities^(22)^. Thus, they may provide key opportunities to reach pregnant women where they are in communities, assist them with insurance enrollment, and facilitate earlier entry into prenatal care.

Clinic-level practices and systems related to scheduling initial appointments were recognized as barriers contributing to delays in entry to care. Similar challenges have been identified in national data. Pregnancy Risk Assessment Monitoring System (PRAMS) data indicated that 38.1% of women reported being unable to secure an appointment, and 27.3% said their provider or health plan would not begin care when needed^(23)^. In our study, communication and scheduling barriers between clinics, patients, and community organizations were evident. Providers described their clinics’ phone systems as difficult to navigate, creating challenges for patients attempting to schedule appointments. As the first point of contact in accessing care, the phone system represents a critical organizational and intrapersonal barrier within the Social Ecological Model (Fig. 1). Additionally, organizations serving pregnant women expressed the need for more direct and efficient ways to secure timely appointments for their patients and clients. This disconnect highlights a broader institutional issue. Implementing user-friendly scheduling systems and providing staff training may help reduce appointment delays.

In our rural areas, the lack of maternity care providers in their own communities and long distances to travel to care posed barriers. In the United States 36% of counties have been referred to as ‘maternity care deserts’; and 56% of rural counties have no maternal care available^(24)^.

Transportation was a major barrier to accessing timely and adequate prenatal care which other researchers have also identified. Pregnant women, individuals with low income, less education, or people of color have been documented as most affected by transportation barriers; longer travel distance may exacerbate challenges and financial burdens, with rural communities facing additional barriers rooted in distance and time ^(25)(24)^. National data show that over 5.8 million people delayed healthcare in 2017 due to transportation difficulties – a trend strongly reflected by our key informants^(25)^.

Across the U.S., women on average travel 9.7 miles to the nearest birthing hospital and have a threshold of 30 to 60 minutes driving to care^(26)^. In our study participants described that travel time for women to prenatal care could be much longer, up to 2 hours each way. Furthermore, lack of any or adequate transportation options was evident. Urban communities also experienced transportation barriers despite relative proximity to care. Our findings corroborate what other studies have shown, that low-income urban neighborhoods often contend with infrastructure barriers and in general the public transportation systems are difficult to navigate^(25)^. Transportation limitations have been directly tied to impacts on healthcare use. One study found that 25% of missed appointments were due to transportation issues, and that patients who rely on public buses are twice as likely to miss appointments^(27)^. Ultimately, our participants also linked transportation struggles to delayed or missed prenatal care. Additional monetary or transportation support, or mobile prenatal services may be explored for improving timely and adequate prenatal care in rural areas.

Distance and transportation to prenatal care must be addressed to improve prenatal care access. Telehealth may be part of the solution to reduce the burden of distance to prenatal care, both during and after pregnancy by reducing geographic disparities. It can be used for a range of services, including prenatal visits, maternal-fetal consultations, chronic illness monitoring, and improving patient-provider engagement^(28)^. With its potential to enhance access and overall health outcomes, effective integration of telehealth could enhance prenatal healthcare delivery.

Our participants noted that many women did not know when, where, or how to access prenatal care. The absence of accessible, community-based educational resources was frequently described as well, leading to confusion and delays in initiating care.

Low health literacy emerged as a major obstacle, particularly among individuals with low education or English proficiency. These findings align with another that showed women with less than a bachelor’s degree received prenatal care later than those with a bachelor’s degree^(23)^. These barriers along with limited preconception education hinder prenatal care in the first-trimester^(29)^.

A broad, community-wide lack of understanding about appropriate timing of prenatal care was identified. Preconception education, provided before a woman becomes pregnant, has been shown to improve pregnancy outcomes and maternal health by identifying and addressing risk factors early^(30)^. It plays a critical role in preparing women for pregnancy and facilitating timely initiation of prenatal care^(31)^. When education is delayed until after pregnancy begins, it may be too late to ensure early prenatal care. Other studies have found that personal beliefs such as the perception that prenatal care was not necessary influenced when women initiated prenatal care^(32)^. Commonly suggested strategies to increase knowledge and education are expanding opportunities for community-based education, increasing social media usage for educational purposes and telehealth in additional languages^(29)^. Additionally, policy makers and community leaders should also be informed about the significance of preconception services to encourage the allocation of resources and integration of prenatal care programs within their communities.

## Limitations

This study has several limitations. Although we made substantial efforts to include a variety of types of informants from each of the 6 counties, there were few healthcare professionals with a maternal health interest in each of the rural counties; thus, although we included at least one healthcare professional from each county, most who participated were concentrated in the two urban counties. This may have limited our understanding about certain rural challenges from a healthcare lens. Additionally, because we included a variety of types participants, we did not include enough participants in any one role to understand differences in perspectives based on roles. For example, it is likely that community leaders, social services personnel, administrators, and healthcare professionals have unique perspectives that differ, which we were not able to ascertain based on this sample. However, we included sufficient numbers of participants in rural counties combined to gain insight into differences between rural and urban contexts; credibility of our findings were validated through member checking and peer-debriefing. By including both rural and urban communities, we obtained a sample that may be similar to other regions in the US. Because of the qualitative approach of this study, our findings may not be generalizable to other contexts.

## Conclusion

Early and consistent prenatal care is essential for preventing complications and managing maternal health conditions. To promote earlier entry into care, community-driven strategies should include support for insurance enrollment to help with applications and ensure timely processing, as well as education on the importance of prenatal care timing and awareness of available resources. Additional strategies may be implemented to addressing long travel distances including improved transportation services, mobile prenatal care units, and strategic telehealth initiatives. Removing barriers within healthcare settings through staff training and updating phone and scheduling systems may also improve timeliness of prenatal care access in underserved communities. Our findings underscore the need to improve and facilitate prenatal care access and implement community-based strategies to reduce gaps in healthcare, education, and resources, particularly among rural and underserved populations.

## Figures and Tables

**Table 1.** Key informant interview guide sample questions

Category	*SEM Level(s)	Example Questions

**Role and Involvement**	Individual	To get started, tell me a little about yourself and your interest/role in maternal health.

	Community	What is your role in identifying, implementing, or providing services?

	Institutional	How does your organization work with other organizations that impact maternal care or services? What are your communication strategies and practices?

**Barriers to Care**	Individual	What are some barriers you think pregnant women may face in receiving prenatal care?

Intrapersonal	What would make going to prenatal visits easier?	
	
Community	Tell me what prenatal care looks like in your community.	
	
**Community Challenges**	Intrapersonal	What sub-population(s) in the community struggle with quality of life?

	Community	Tell me about some challenges of your community?

	Societal/Policy	What efforts have been made to address these challenges.

**Risk Factors**	Individual	What are some circumstances that increase someone’s risk?

	Intrapersonal	Who is at greater risk for pregnancy complications in your community?

	Community	Do you think women in your community know how to reduce their risk of having complications during their pregnancy?

**Needs**	Individual	In what ways do you think pregnant women need more support in your community?

	Intrapersonal	What resources would be helpful during pregnancy that are not available?

	Societal/Policy	What resources or programs do you think would help combat pregnancy and maternal health problems?

* SEM Levels = Social Ecological Model Levels

**Table 2:** Key informant demographics and characteristics.

	Median	Range
**Age**	49.0	32–70
	n	%
**Sex**		
Female	29	85.3
Male	5	14.7
**Race**		
White	22	64.7
Hispanic	8	23.5
Black	3	8.8
Asian	1	2.9
**Ethnicity**		
Hispanic	10	29.4
Non-Hispanic	24	70.6
**Education**		
Professional degree	14	41.2
Bachelor’s degree	12	35.3
Some College	4	11.8
Associate’s degree	3	8.8
High School	1	2.9
**Occupation**		
Social Services	11	32.4
Community Leaders	5	14.7
Administrators	5	14.7
Healthcare Professionals	13	38.2
**County**		
Randall	10	29.4
Potter	6	17.6
Deaf Smith	5	14.7
Swisher	4	11.8
Gray	4	11.8
Parmer	5	14.7

**Table 3:** Themes, subthemes, and example quotations related to contributors to delayed prenatal care.

Themes	Subthemes	Example Quotations
Lack of insurance	Delayed insurance application approvals	Hard time applying or not necessarily applying for Medicaid, but getting their approvals from Medicaid in a timely manner that they’re not getting prenatal care until a lot later, because they can’t get Medicaid. (KI120)
	Limited services for low-income women	*Umm* again, has to do with not having insurance. *Umm* some of the lower scale Medicare or Medicaid do not always provide the full service that a private insurance does. (KI102)
	Insurance coverage gaps	They don’t have health insurance… even if they have a health issue, they can’t get taken care of because they don’t have any way to pay for that health care. (KI103)
	Limited access to internet and technology	I know some of our families …apply online for Medicaid. They don’t always have the… technological…capability or understanding to go through and complete the application. (KI104)
Healthcare setting challenges	Limited providers for low-income women	Well… there’s a lot of doctors that won’t even take Medicaid. So that’s a barrier right from the start. They do have to have knowledge of a doctor that will accept their Medicaid. (KI113)
	Appointment scheduling barriers	I’ve heard women don’t receive prenatal care early for several different reasons. Either it’s hard to get on Medicaid, or the physician, the OB doctor, doesn’t take them until after they’re 12 weeks pregnant. And then I’ve also heard they’ve had a hard time getting an appointment for prenatal care. (KI115)
Distance and transportation to prenatal care	Long travel distances	Transportation is a huge deal. Gas is expensive for those that do have a vehicle and going to [a larger city] is actually an all-day thing, it takes you an hour and a half to get there… and you might be in your appointment two hours…and then you got to drive back…so you’ve just spent six hours going to one appointment and that takes your day. (KI109)
	Urban communities’ proximity to care	South and west of us… that’s where the doctor’s offices go. That’s where the hospitals are. That’s where the resources are. It’s like our north and east neighborhoods have been left behind … if you don’t have transportation to get to [the healthcare provider], what’s your other choice if you live in the [neighborhoods with low income]? There’s no choices. (KI103)
	Inadequate transportation options	The bus system here is not great… and I know Medicaid pays for [rides], but it’s a complicated process. (KI103)
Gaps in knowledge and understanding about prenatal care	Low health literacy	Well, and first of all, a lot of our low-income families their education levels are low, so they may work in jobs that are lower paid, so they they’re struggling with poverty. *Umm* they are struggling with low education level itself leads to not really understanding health or what a doctor tells you, so poor health literacy. (KI119)
	Lack of education for teens and pregnant adolescents	There should be… in every school starting in the middle schools and the high school, there should be consistent support there, there’s a lot of young moms that are, they get pregnant and they don’t know what to do. They never had education about, hey, what happens if I get pregnant? So, they try to hide it, they try to -- it’s stigmatized. So, I feel like, really good programs, especially starting in the schools would be great. (KI113)
	Lack of pre-pregnancy education	I think women don’t necessarily know how to have a healthy pregnancy. I think all they focus on is having the baby, but not understanding how important it is to take care of themselves while the baby is in the womb. (KI101)
	Community wide education	Not having the education. You need to know what to do once you are pregnant. The dads need education just as much as the [pregnant moms]. (KI113)

## Data Availability

The datasets generated and/or analyzed during the current study are available from the corresponding author on reasonable request. Only de-identified excerpts from the interviews will be shared to protect participant confidentiality.

## Abbreviations

ACOG: American College of Obstetricians and Gynecologists
CFIR: Consolidated Framework for Implementation Research
CHWs: Community Health Workers
KI: Key Informant
PRAMS: Pregnancy Risk Assessment Monitoring System
SEM: Social Ecological Model
US: United States
